# Circular RNA 0006349 Augments Glycolysis and Malignance of Non-small Cell Lung Cancer Cells Through the microRNA-98/MKP1 Axis

**DOI:** 10.3389/fcell.2021.690307

**Published:** 2021-09-17

**Authors:** Chu Qin, Rongguo Lu, Minyu Yuan, Rui Zhao, Huiya Zhou, Xiaodong Fan, Bo Yin, Haoda Yu, Tao Bian

**Affiliations:** ^1^Department of Respiratory Medicine, Wuxi People’s Hospital, Wuxi, China; ^2^Department of Thoracic Surgery, Wuxi People’s Hospital, Wuxi, China; ^3^Department of Respiratory Medicine, Wuxi No. 8 People’s Hospital, Wuxi, China

**Keywords:** Circ_0006349, miR-98, MKP1, NSCLC, proliferation, glycolysis

## Abstract

**Background:** The involvement of dysregulated circular RNAs (circRNAs) in human diseases has been increasingly recognized. In this study, we focused on the function of a newly screened circRNA, circ_0006349, in the progression of non-small-cell lung cancer (NSCLC) and the molecules of action.

**Methods:** The NSCLC circRNA dataset GSE101684, microRNA (miRNA) dataset GSE29250, and mRNA dataset GSE51852 obtained from the GEO database were used to identify the differentially expressed genes in NSCLC samples. Tumor and normal tissues were collected from 59 patients with NSCLC. The expression of circ_0006349, miR-98, and MAP kinase phosphatase 1 (MKP1) in collected tissue samples and in acquired cells was determined. The binding relationships between miR-98 and circ_0006349/MKP1 were predicted and validated. Altered expression of circ_0006349, miR-98, and MKP1 was introduced in NSCLC cells to examine their roles in cell growth, apoptosis, and glycolysis.

**Results:** Circ_0006349 and MKP1 were upregulated, and miR-98 was poorly expressed in the collected tumor tissues and the acquired NSCLC cell lines. Circ_0006349 was identified as a sponge for miR-98 to elevate MKP1 expression. Silencing of circ_0006349 suppressed proliferation and increased apoptosis of Calu-3 and H1299 cells, and it reduced glycolysis, glucose uptake, and the production of lactate in cells. Upon circ_0006349 knockdown, further downregulation of miR-98 or upregulation of MKP1 restored the malignant behaviors of cells.

**Conclusion:** This research demonstrated that circ_0006349 derepressed MKP1 expression by absorbing miR-98, which augmented the proliferation and glycolysis of NSCLC cells and promoted cancer development.

## Introduction

Lung cancer (LC) remains a leading cause of cancer morbidity and mortality worldwide, with an estimated 2,093,876 cases and 1,761,007 deaths in 2018 globally, representing almost one-fifth of all cancer deaths (18.4%) (Bray et al., 2018). Approximately 85% of LC cases are non-small cell LCs (NSCLCs), and this subgroup further encompasses several cancer types, such as adenocarcinomas, squamous cell cancers, and large cell cancers (Relli et al., 2019). Tobacco smoking represents the major risk factor for this disease, and air pollution or radon exposure may also play a role (Gridelli et al., 2015). It was reported that two-thirds of patients are diagnosed with metastatic lesions, and metastasis and recurrence bring a less than 20% 5-year survival rate in these patients even following conventional therapies, including surgical resection, chemotherapy, and radiotherapy (Guo et al., 2020; Lai et al., 2020). Hence, developing novel options for NSCLC management remains an emergent issue with great significance.

One of the essential characteristics of cancers is the abnormal proliferation of cells, which is always attributed to aberrant energy metabolism (Choi et al., 2013). Aerobic glycolysis, rather than oxidative phosphorylation, is preferred for energy metabolism in cancer cells and is termed the “Warburg effect,” promoting lactate production, proton accumulation, and the subsequent invasive and proliferative tendencies of these cells (Koppenol et al., 2011; Feng and Yang, 2020). Controlling glycolysis and dysregulated proliferation may serve as a therapeutic target in cancer management.

Over 75% of the human genome is transcribed into RNAs, and these RNAs, including protein-encoding RNAs and a larger class of noncoding RNAs (ncRNAs), interact and exert versatile functions in disease progression (Dai et al., 2020). MicroRNAs (miRNAs/miRs) represent a superfamily of small ncRNAs that govern gene expression in cells post-transcriptionally, and their aberrant expression is frequently observed in cancers, including NSCLC (Petrek and Yu, 2019). Despite binding to mRNAs, miRNAs can also bind to other ncRNAs, including long ncRNAs (lncRNAs) and circular RNAs (circRNAs), termed competitive endogenous RNAs (ceRNAs), and thus ncRNAs may compete with a limited number of miRNAs through shared miRNA response elements (Salmena et al., 2011; Karreth and Pandolfi, 2013). CircRNAs, a unique type of lncRNA with a closed loop structure (Shabaninejad et al., 2019), have been increasingly found to have essential roles in NSCLC development through such ceRNA networks (Wang et al., 2020; Yang et al., 2020). In the present study, with the aid of NSCLC-related GSE datasets from the Gene Expression Omnibus (GEO) database, circ_0006349 was identified as a candidate regulator of NSCLC since it was significantly upregulated in NSCLC samples. However, there is no study to date concerning its correlation with human diseases. The following bioinformatic analyses predicted that this circRNA possibly absorbs miR-98 to upregulate MAP kinase phosphatase 1 (MKP1). Increased miR-98 expression induced by aspirin has been recently reported to ameliorate LC (Gan et al., 2019). MKP1, also called dual-specificity phosphatase-1 (DUSP1), is a member of the threonine-tyrosine dual-specificity phosphatase family that governs the activity of proliferation- or apoptosis-related pathways and plays key roles in tumorigenesis (Shen et al., 2016). Therefore, we surmised that circ_0006349 possibly interacts with miR-98 and MKP1 and participates in NSCLC progression. Gain- and loss-of function studies of these molecules were performed to validate this hypothesis.

## Materials and Methods

### Bioinformatic Analyses

The circRNA dataset GSE101684, miRNA dataset GSE29250, and mRNA dataset GSE51852 of NSCLC were obtained from the GEO database. The data were normalized and analyzed using the Limma R Package.^1^ The criteria for gene screening were |Log FC| > 1 and adjusted *p* value < 0.05. The heatmaps were produced by an R pheatmap package.^2^ The potential target miRNAs of circ_0006349 were predicted using the circBank browser,^3^ while the target mRNAs of miR-98 were predicted using StarBase^4^ and TargetScan.^5^ Venn diagrams were produced using the Venn browser.^6^

### Sample Collection

From July 2018 to June 2019, a total of 59 patients with primary NSCLC who were treated in Wuxi People’s Hospital were recruited for this study. All patients were free of any other malignancies and without a history of radiotherapies or chemotherapies. Tumor tissues and adjacent normal tissues from patients were collected during surgery, bronchoscopic biopsy, or percutaneous lung puncture. Samples were instantly frozen in liquid nitrogen and preserved at −80°C for further use. This study was approved by the Ethics Committee of Wuxi People’s Hospital and in line with the Declaration of Helsinki. Informed consent was obtained from each participant.

### Reverse Transcription Quantitative Polymerase Chain Reaction

Total RNA from tissues and cells was extracted using TRIzol^®^ Reagent (Invitrogen, Thermo Fisher Scientific Inc., Waltham, MA, United States). The RNA was reverse-transcribed into cDNA using M-MLV buffer, dNTPs and random primers, and a Moloney murine leukemia virus RT kit (all from Promega Corp., Madison, Wisconsin, United States) according to the manufacturer’s instructions at 37°C for 60 min. Next, qPCR was conducted using SYBR Green Real-time PCR Master Mix (Solarbio Science and Technology Co., Ltd., Beijing, China) on a Bio–Rad CFX96 System (BioRad, Berkeley, CA, United States). Relative gene expression was determined using the 2^–DDCq^ method (Livak and Schmittgen, 2001). The primer sequences are listed in Table 1, and GAPDH and U6 were used as internal controls.

**TABLE 1:** Primer sequences for RT-qPCR.

Gene	Primer sequence (5′-3′)
Circ_0006349	F: TTTGGGATGTACATTCCGTT
	R: TCCCACTTCCGCTTCCA
MKP1	F: CAACCACAAGGCAGACATCAGC
	R: GTAAGCAAGGCAGATGGTGGCT
miR-98	F: GAGGTAGTAAGTTGTATTG
	R: GAACATGTCTGCGTATCTC
U6	F: CTCGCTTCGGCAGCACAT
	R: TTTGCGTGTCATCCTTGCG
GAPDH	F: GTCTCCTCTGACTTCAACAGCG
	R: ACCACCCTGTTGCTGTAGCCAA

*RT-qPCR, reverse transcription quantitative polymerase chain reaction; MKP1, MAP kinase phosphatase 1; miR, microRNA; GAPDH, glyceraldehyde-3-phosphate dehydrogenase; F, forward; R, reverse.*

### Cell Culture and Transfection

The human bronchial epithelial cell line BEAS-2B, six NSCLC cell lines (A549, H1299, Calu-3, H520, H1650, and H1730), and HEK-293T cells were acquired from the Cell Bank of the Chinese Academy of Sciences (Shanghai, China). HEK-293T cells were cultured in Dulbecco’s modified Eagle’s medium (DMEM, Gibco Company, Grand Island, NY, United States), whereas the remaining cell lines were cultivated in Roswell Park Memorial Institute (RPMI)-1640 (Gibco) supplemented with 10% foetal bovine serum (FBS) at 37°C with 5% CO_2_. Exponentially growing cells were used for further experiments.

In subsequent experiments, Calu-3 and H1299 cells were transfected with small interfering RNAs (siRNAs) targeting circ_0006349, miR-98 inhibitor, or overexpression vector of MKP1 (oe-MKP1). Transfection was performed using Lipofectamine 2000 reagent (Invitrogen) according to the manufacturer’s instructions. The transfection efficacy was examined by RT–qPCR 48 h later.

### 5-Ethynyl-2′-Deoxyuridine Labeling Assay

Cells seeded in 96-well plates were incubated with 100 μL EdU reagent (Solarbio) for 2 h. The cells were washed with phosphate-buffered saline (PBS) and stained with Apollo reagent. Next, the cells were stained with methanol, fixed, permeated, and then cultured with 100 μL 4′,6-diamidino-2-phenylindole (DAPI) reaction solution (Sigma–Aldrich, Merck KGaA, Darmstadt, Germany). Positive EdU labeling, namely, the viability of cells, was determined under a fluorescence microscope (Thermo Fisher Scientific).

### Flow Cytometry

An Annexin V-fluorescein isothiocyanate (FITC) apoptosis detection kit (BD Pharmingen, San Diego, CA, United States) was used to measure cell apoptosis. In brief, at 48 h after transfection, cells were washed, cleaned, and incubated with 5 μL Annexin V-FITC and 5 μL propidium iodide (PI) in the dark for 15 min of double staining. Cell apoptosis was determined using a FACScan flow cytometer (Becton Dickinson, Mountain View, CA, United States).

### Determination of Extracellular Acidification Rate in Cells

Extracellular Acidification Rate, a reflection of glycolysis activity in cells, was determined by a Seahorse Extracellular Flux Analyser XF96 (Seahorse Bioscience, North Billerica, MA, United States). Briefly, 2 × 10^4^ cells were cultured in 96-well plates overnight. The cells were successively incubated with glucose, oligomycin (an oxidative phosphorylation inhibitor), and 2-deoxyglucose (2-DG, a glycolysis inhibitor). The data were analyzed using Seahorse XF-96 Wave software. The ECAR was presented as mpH/min.

### Determination of Glucose Uptake and Lactate Production

A glucose uptake colorimetric assay kit (BioVision, Milpitas, CA, United States) was used to determine glucose uptake in cells. In short, cells were seeded in 6-well plates at 1 × 10^4^ cells per well and incubated in 100 mL Krebs-Ringer-Phosphate-Hepes (KRPH) buffer containing 2% bovine serum albumin, followed by further addition of 10 mM 2-DG. After that, the samples were collected for glucose uptake measurement. For lactate detection, cells were seeded in 96-well plates and starved in serum-free medium for 24 h. Then, the cells were suspended in lactate detection kit II buffer (BioVision, Exton, PA, United States). All measurements, using the glucose uptake colorimetric assay kit and the lactate detection kit II, were performed in line with the manufacturer’s instructions.

### Colony Formation Assay

After transfection, cells were incubated in 6-well plates in DMEM containing 10% FBS. Two weeks later, the cells were fixed in methanol for 30 min and stained with 1% crystal violet dye. The number of cell colonies was counted under a microscope (Olympus, Optical Co., Ltd, Tokyo, Japan).

### Terminal Deoxynucleotidyl Transferase-Mediated dUTP Nick End Labeling

Apoptosis of cells was examined using a TUNEL kit (Beyotime Biotechnology Co. Ltd., Shanghai, China) according to the manufacturer’s instructions. The results were observed and analyzed using a confocal laser scanning microscope (Leica Microsystems CMS GmbH Am Friedensplatz 3, 68165 Mannheim Germany).

### Western Blot Analysis

Protein samples were collected, and the protein concentration was examined using the bicinchoninic acid assay. Next, an equal volume of protein (30 μg) was electrophoresed on an 8% –10% SDS–PAGE gel and then loaded onto nitrocellulose membranes (Millipore, Billerica, MA, United States). Membranes were blocked in 5% nonfat milk for 1 h and incubated with rabbit antibody against human MKP1 (dilution 1:1,000; Abcam, Cambridge, United Kingdom) and β-actin antibody overnight at 4°C. After being washed in PBS, each membrane was incubated with a peroxidase-conjugated secondary antibody at 25°C for 1–2 h. The protein bands were developed with ECL reagent (Millipore). The density of each band was evaluated using Image-Pro Plus 6.0 software.

### Nuclear-Cytoplasmic RNA Separation

A nuclear-cytoplasmic RNA purification kit (Norgen Biotek Corp., Belmont, MA, United States) was utilized to examine the subcellular localization of hsa_circ_0006349 in cells. First, cells were lysed in lysis buffer, and then the lysates were centrifuged. After that, the nuclear and cytoplasmic RNA was loaded in absolute ethanol and buffer SK, respectively. The RNA was eluted in a cyclone column, and the concentration of hsa_circ_0006349 in the nucleus and cytoplasm was determined by RT-qPCR.

### Fluorescence *in situ* Hybridization

The Cy3 labeling probe of hsa_circ_0006349 was designed by GenePharma Co., Ltd. (Shanghai, China). The subcellular localization of hsa_circ_0006349 in cells was further determined using an RNA FISH kit (GenePharma) according to the manufacturer’s instructions. DAPI was used for counter-staining. FISH images were captured under a confocal microscope (Olympus).

### Dual-Luciferase Reporter Gene Assay

Psicheck2-based vectors (Promega) containing the wild-type (WT) sequence of circ_0006349 or the 3′-UTR of MKP1 mRNA were constructed (defined as psicheck2-WT-hsa_circ_0006349, psicheck2-WT-MKP1, or psicheck2-control). Vectors based on mutant-type (MT) sequences were constructed as well. Well-constructed WT or MT vectors were cotransfected with either miR-98 mimic or miR-98 inhibitor (GenePharma) into 293T cells. After 24 h, the relative luciferase activity in cells was determined using a dual-luciferase determination system (Promega) based on the firefly and Renilla luciferase activities.

### RNA Immunoprecipitation by Anti-Ago2

The RIP assay was performed using an EZ Magna RIP kit (Millipore) according to the manufacturer’s instructions. The cells were lysed in complete RIP lysis buffer. The extracts were incubated with anti-Ago2 or anti-IgG (Millipore) at 4°C for 6 h, followed by the addition of protein A/G (Thermo Fisher Scientific)-conjugated magnetic beads. The beads were washed and incubated with protease K to remove the protein. Then, the relative expression of purified circ_0006349 or MKP1 in the complexes was determined by RT-qPCR.

### Statistical Analysis

All data are presented as the mean ± standard deviation (SD) from three independent experiments. Prism 5.0 (GraphPad, Software, San Diego, CA, United States) was used for statistical analysis. Differences were analyzed by Student’s *t* test (between two groups) or analysis of variance (ANOVA, among multiple groups). Correlations between variables were compared by Pearson’s correlation analysis. ^∗^*p* < 0.05 was considered as statistically significant in general.

## Results

### Circ_0006349 Is Highly Expressed and Indicates Dismal Prognosis in Patients With NSCLC

**FIGURE 1:**
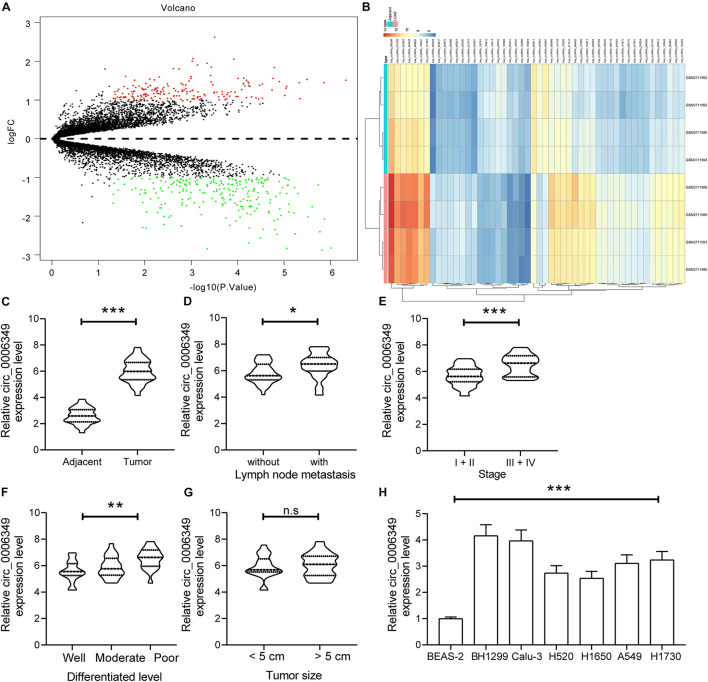
Circ_0006349 is highly expressed and indicates dismal prognosis in patients with non-small-cell lung cancer (NSCLC). **(A)** A volcano map for circRNAs screened out from the NSCLC GSE101684 dataset; **(B)** a heatmap for top 50 differentially expressed circRNAs in the GSE101684 dataset; **(C)** circ_0006349 expression in tumor and normal tissues from 59 patients with NSCLC determined by reverse transcription quantitative polymerase chain reaction (RT-qPCR); **(D–G)** relevance between circ_0006349 expression with the lymph node metastasis, tumor stage, tumor differentiation and tumor size in patients; **(H)** circ_0006349 expression NSCLC cell lines (A549, H1299, Calu-3, H520, H1650, and H1730) and in BEAS-2B cells detected by RT-qPCR. Differences were compared by paired *t* test **(C)**, unpaired *t* test **(D–G)**, or one-way ANOVA **(H)**. All data were presented as the mean ± SD from three independent experiments. *p < 0.05; **p < 0.01; ***p < 0.001; n.s, no significance.

A circRNA dataset GSE101684 comprising data on tumor and paratumor tissues from 4 patients with lung adenocarcinoma was downloaded from the GEO database. Using the Limma R Package, a total of 415 differentially expressed circRNAs were screened in the settings of |Log FC| > 1 and adjusted *p* value < 0.05 (Figure 1A). The top 50 differentially expressed genes are listed in a heatmap in Figure 1B. Among them, the expression of hsa_circRNA_0006349 was significantly increased in the tumor tissues compared to the normal tissues. Thereafter, the expression of circ_0006349 was detected in the collected tumor tissues and the paired normal tissues from 59 NSCLC patients. The RT-qPCR results suggested that circ_0006349 expression was significantly increased in tumor tissues (Figure 1C). Furthermore, the correlations between circ_0006349 expression and lymph node metastasis, tumor stage, tumor size, and tumor differentiation in patients were determined. Patients with positive lymph node metastasis had higher circ_0006349 expression than those without metastasis (Figure 1D). In addition, increased expression of circ_0006349 was associated with advanced tumor grades and poor tumor differentiation (Figures 1E,F), whereas no significant correlation was found between circ_0006349 expression and tumor size (Figure 1G). Furthermore, we determined circ_0006349 expression in acquired NSCLC cell lines (A549, H1299, Calu-3, H520, H1650, and H1730) and BEAS-2B cells. Nevertheless, increased expression of circ_0006349 was found in all NSCLC cell lines compared to BEAS-2B cells (Figure 1H).

### Silencing of circ_0006349 Suppresses the Malignant Behaviors of NSCLC Cells

**FIGURE 2:**
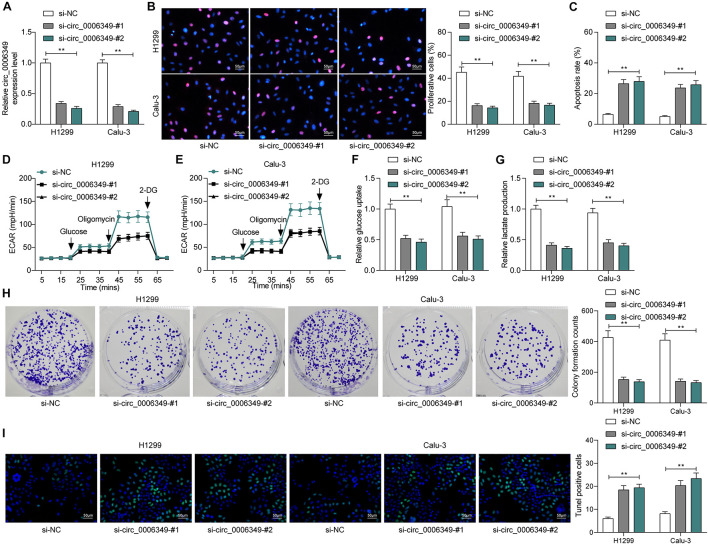
Silencing of circ_0006349 suppresses the malignant behaviors of NSCLC cells. **(A)** transfection efficacy of si-circ_0006349-#1 and si-circ_0006349-#2 in H1299 and Calu-3 cells determined by RT-qPCR; **(B)** DNA replication activity of H1299 and Calu-3 cells measured by EdU labeling assay; **(C)** apoptosis of H1299 and Calu-3 cells detected by the flow cytometry; **(D,E)** ECAR in H1299 and Calu-3 cells; **(F,G)** glucose uptake **(F)** and lactate production **(G)** in H1299 and Calu-3 cells; **(H)** colony formation ability of H1299 and Calu-3 cells examined by the colony formation assay; **(I)** apoptotic bodies in H1299 and Calu-3 cells examined by the TUNEL assay. Differences were compared by two-way ANOVA **(A–C,F–I)**. All data were presented as the mean ± SD from three independent experiments. **p < 0.01.

To identify the possible functions of circ_0006349 in the behaviors of NSCLC cells, two siRNAs targeting circ_0006349 (si-circ_0006349-#1 and si-circ_0006349-#2) were introduced into H1299 and Calu-3 cells, which had the highest circ_0006349 expression among the five NSCLC cell lines. The transfection efficacy was confirmed by RT-qPCR (Figure 2A). Thereafter, the EdU labeling assay suggested that the DNA replication activity of H1299 and Calu-3 cells was decreased (Figure 2B), and the flow cytometry results showed that the number of apoptotic cells was increased (Figure 2C) upon circ_0006349 silencing. In addition, the ECAR in cells was further investigated. In the setting of circ_0006349 silencing, glycolysis in H1299 and Calu-3 cells was notably decreased (Figures 2D,E), and glucose uptake and lactate production in cells were reduced (Figures 2F,G). Likewise, the colony formation assay suggested that silencing circ_0006349 reduced the number of colonies formed by H1299 and Calu-3 cells (Figure 2H). The TUNEL assay suggested that the number of apoptotic bodies in cells was significantly increased upon circ_0006349 knockdown (Figure 2I).

### Circ_0006349 Is Sublocalized in the Cytoplasm and Binds to miR-98

**FIGURE 3:**
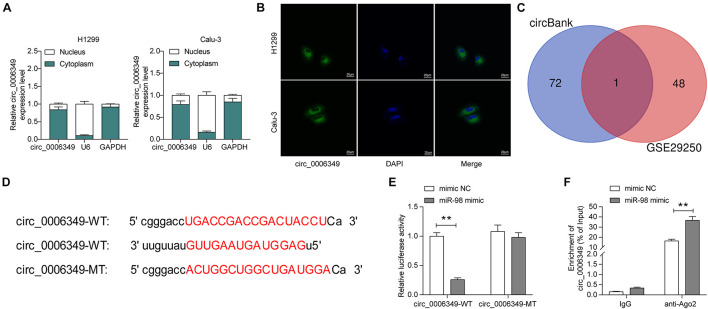
Circ_0006349 sub-localizes in cytoplasm and binds to miR-98. **(A,B)** subcellular localization of circ_0006349 in Calu-3 and H1299 cells determined by nuclear-cytoplasmic RNA separation **(A)** and FISH **(B)** assays; **(C)** a Venn diagram for the intersection of screened out differentially expressed miRNAs from the GSE29250 dataset and the predicted target miRNAs of circ_0006349; **(D)** putative binding site between circ_0006349 and miR-98; **(E,F)** binding relationship between miR-98 and circ_0006349 validated through dual luciferase reporter gene and RIP assays. Differences were compared by two-way ANOVA **(E,F)**. All data were presented as the mean ± SD from three independent experiments. **p < 0.01.

The focus of the study then shifted to the molecular mechanism involved in the above events. First, the subcellular localization of circ_0006349 was determined. Either the nuclear-cytoplasmic RNA separation or the FISH assay indicated that circ_0006349 was mainly sub-localized in the cytoplasm (Figures 3A,B). The NSCLC miRNA dataset GSE29250 containing data from 6 pairs of tumor and normal tissues was obtained from the GEO database, in which a total of 48 differentially expressed miRNAs were identified. These miRNAs were compared with the potential binding miRNAs of circ_0006349 predicted on the circBank browser, and miR-98 was found to intersect (Figure 3C). To further explore the binding relationship between miR-98 and circ_0006349, a dual-luciferase reporter assay was performed, which showed that cotransfection of miR-98 and circ_0006349-WT led to a notable decline in luciferase activity in 293T cells (Figures 3D,E). In addition, an RIP assay was performed in which miR-98 mimic or mimic NC was transfected into 293T cells, and the cells were further incubated with anti-Ago2 for immunoprecipitation. In the complexes precipitated by anti-Ago2, circ_0006349 expression was increased in cells transfected with miR-98, while no significant difference was observed in the complexes precipitated by anti-IgG (Figure 3F). These results indicated that circ_0006349 could directly bind to miR-98.

### miR-98 Is Poorly Expressed in NSCLC and Presents a Negative Correlation With circ_0006349

**FIGURE 4:**
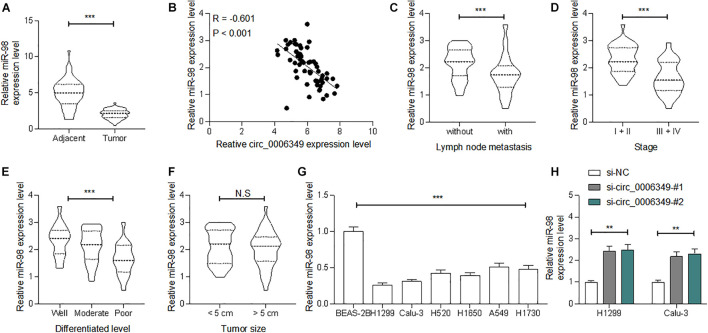
miR-98 is poorly expressed in NSCLC and presents a negative correlation with circ_0006349. **(A)** miR-98 expression in tumor and normal tissues from 59 patients with NSCLC determined by RT-qPCR; **(B)** a reverse correlation between miR-98 and circ_0006349 expression in tumor tissues analyzed by Pearson’s correlation test; **(C–F)** relevance between miR-98 expression with the lymph node metastasis, tumor stage, tumor differentiation and tumor size in patients; **(G)** miR-98 expression in NSCLC cell lines (A549, H1299, Calu-3, H520, H1650, and H1730) and BEAS-2B cells detected by RT-qPCR. Differences were compared by paired *t* test **(C)**, unpaired *t* test **(C–F)**, one-way **(G)** or two-way ANOVA **(H)**. All data were presented as the mean ± SD from three independent experiments. **p < 0.01; ***p < 0.001; n.s, no significance.

Following the findings above, we determined miR-98 expression in the collected tumor tissues and adjacent normal tissues from the 59 patients. The RT-qPCR results showed that miR-98 was poorly expressed in tumor tissues (Figure 4A), which showed an inverse correlation with circ_0006349 (Figure 4B). High miR-98 expression in patients was correlated with decreased lymph node metastasis and tumor grade as well as advanced tumor differentiation, whereas it showed no significant impacts on tumor size (Figures 4C–F). In cells, miR-98 expression was decreased in NSCLC cell lines compared to BEAS-2B cells (Figure 4G). As expected, downregulation of circ_0006349 led to an increase in miR-98 expression in H1299 and Calu-3 cells (Figure 4H).

### miR-98 Inhibitor Rescues the Malignant Behaviors of NSCLC Cells

**FIGURE 5:**
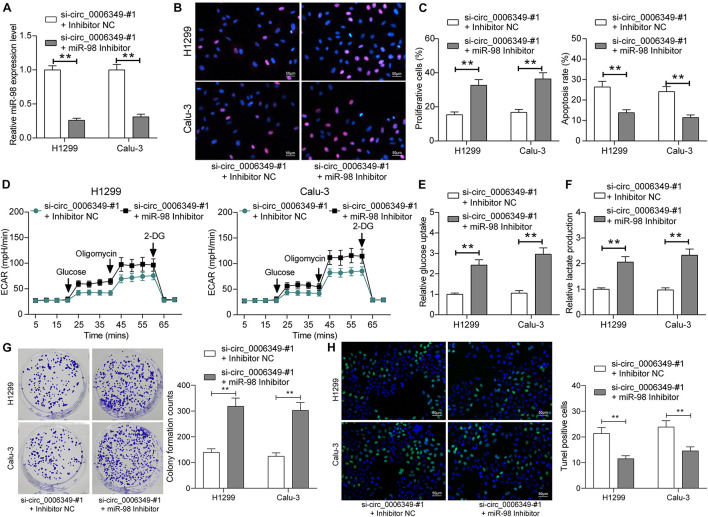
miR-98 inhibitor promotes the malignant behaviors of NSCLC cells. **(A)** Transfection efficacy of miR-98 in H1299 and Calu-3 cells determined by RT-qPCR; **(B)** DNA replication activity of H1299 and Calu-3 cells measured by EdU labeling assay; **(C)** apoptosis of H1299 and Calu-3 cells detected by the flow cytometry; **(D)** ECAR in H1299 and Calu-3 cells; **(E,F)** glucose uptake **(E)** and lactate production **(F)** in H1299 and Calu-3 cells; **(G)** colony formation ability of H1299 and Calu-3 cells examined by the colony formation assay; **(H)** apoptotic bodies in H1299 and Calu-3 cells examined by the TUNEL assay. Differences were compared by two-way ANOVA **(A–C,E–H)**. All data were presented as the mean ± SD from three independent experiments. **p < 0.01.

To confirm the function of miR-98 in this disease, a miR-98 inhibitor was additionally administered to H1299 and Calu-3 cells pretransfected with si-circ_0006349. Again, the transfection efficacy was validated by RT-qPCR (Figure 5A). Thereafter, it was found that the proliferation activity of cells suppressed after circ_0006349 silencing was restored after miR-98 inhibition (Figure 5B), and the apoptosis of H1299 and Calu-3 cells was blocked (Figure 5C). Importantly, the reduced ECAR, glycolysis, glucose uptake, and lactate production in cells suppressed upon circ_0006349 silencing were rescued by the miR-98 inhibitor (Figures 5D–F). Likewise, the number of cell colonies formed by cancer cells was significantly increased by the miR-98 inhibitor (Figure 5G), and the TUNEL-positive rate in cells increased by si-circ_0006349 was blocked after further miR-98 inhibition (Figure 5H).

### miR-98 Directly Binds to MKP1 mRNA

**FIGURE 6:**
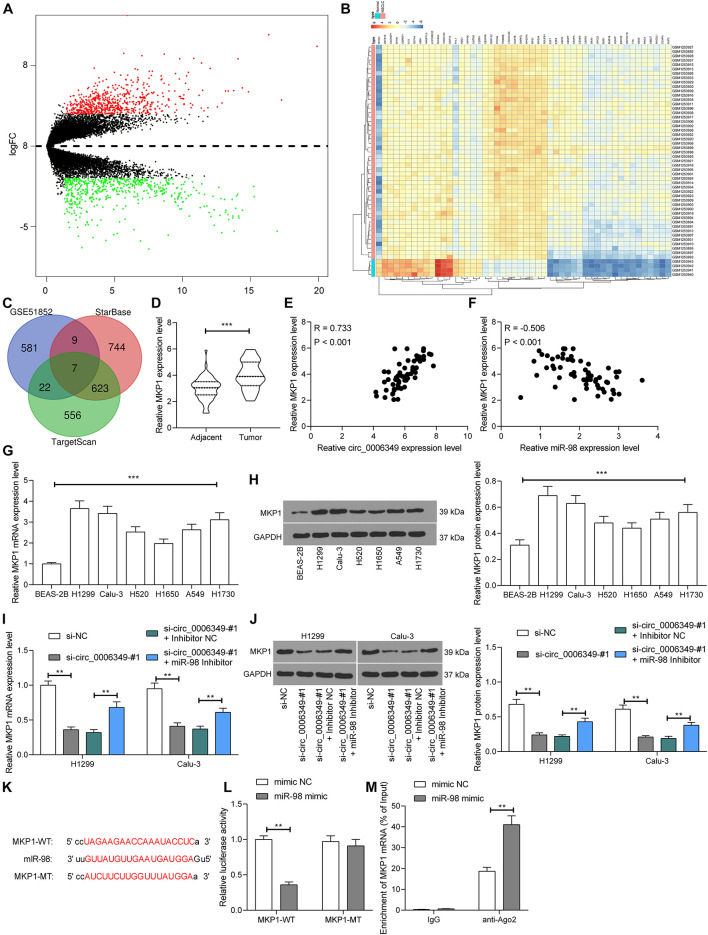
miR-98 directly binds to MKP1 mRNA. **(A)** Volcano plots for the differentially expressed mRNAs in the GSE51852 dataset; **(B)** a heatmap for to 50 differentially expressed mRNAs in the GSE51852 dataset; **(C)** a Venn diagram for the intersections of target mRNAs of miR-98 and the differentially expressed mRNAs in NSCLC; **(D)** mRNA expression of MKP1 in tissue and normal tissues in 59 NSCLC patients determined by RT-qPCR; **(E)** a positive correlation between MKP1 expression and circ_0006349 in tumor tissues according to the Pearson’s correlation test; **(F)** a negative correlation between MKP1 expression and miR-98 in tumor tissues according to the Pearson’s correlation test; **(G,H)** mRNA **(G)** and protein **(H)** expression of MKP1 in BEAS-2B and NSCLC cells determined by RT-qPCR and western blot analysis, respectively; **(I,J)** mRNA **(I)** and protein **(J)** expression of MKP1 in Calu-3 and H1299 cells after si-circ_0006349 or miR-98 inhibitor transfection determined by RT-qPCR and western blot analysis, respectively; **(K)** sequences of the MKP1-WT and MKP1-MTL vectors for luciferase assay; **(L,M)** binding relationship between miR-98 and MKP1 mRNA validated through dual luciferase reporter gene **(L)** and anti-Ago2-RIP **(M)** assays. Differences were compared by paired *t* test **(D)**, one-way **(G,H)** or two-way ANOVA **(I,J,L,M)**. All data were presented as the mean ± SD from three independent experiments. **p < 0.01; ***p < 0.001.

Next, the NSCLC mRNA dataset GSE51852 was obtained from the GEO dataset, which comprised data from 4 normal lung tissue samples and 49 NSCLC tissue samples. A total of 2024 differentially expressed mRNAs were screened in the settings of |Log FC| > 2 and adjusted p value < 0.05 (Figure 6A). The heatmap in Figure 6B lists the top 50 differentially expressed genes. Then, we explored the possible targeting mRNAs of miR-98 using the browsers StarBase and TargetScan. The outcomes were compared with the above aberrantly expressed mRNAs in NSCLC. Among the intersecting mRNAs, MKP1 aroused our attention, since it has been associated with increased resistance to cisplatin-induced apoptosis in NSCLC cells (Chattopadhyay et al., 2006). Thereafter, MKP1 expression in 59 pairs of tumor and normal tissues collected from NSCLC patients was determined. The RT-qPCR results suggested that MKP1 had higher expression in tumor tissues than in normal tissues (Figure 6D). Intriguingly, in cancer tissues, MKP1 expression showed a positive correlation with circ_0006349 and a negative correlation with miR-98 (Figures 6E,F). Similarly, in cells, increased expression of MKP1 was identified in NSCLC cells compared to BEAS-2B cells (Figures 6G,H). In H1299 and Calu-3 cells, silencing circ_0006349 led to a decline in MKP1 expression, whereas further administration of the miR-98 inhibitor re-enhanced the level of MKP1 (Figures 6I,J). The following dual-luciferase reporter gene and RIP assays validated a direct binding relationship between miR-98 and the 3′-UTR of MKP1 (Figures 6K–M).

### Overexpression of MKP1 Rescues the Malignant Behaviors of NSCLC Cells Inhibited by si-circ_0006349

**FIGURE 7:**
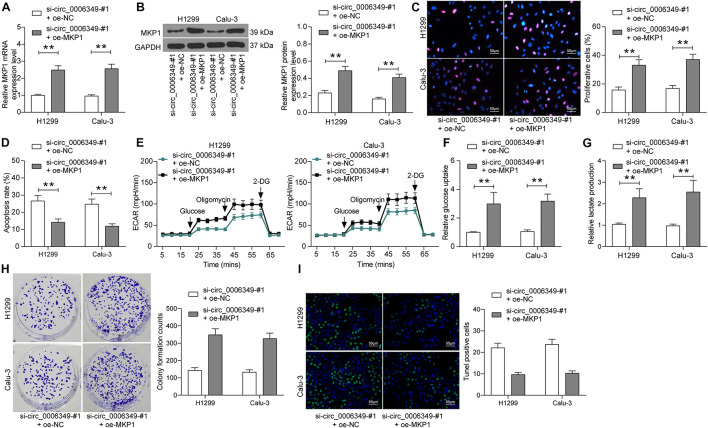
Overexpression of MKP1 promotes the malignant behaviors of NSCLC cells. **(A,B)** transfection efficacy of oe-MKP1 in H1299 and Calu-3 cells validated by RT-qPCR and western blot analysis, respectively; **(C)** DNA replication activity of H1299 and Calu-3 cells measured by EdU labeling assay; **(D)** apoptosis of H1299 and Calu-3 cells detected by the flow cytometry; **(E)** ECAR in H1299 and Calu-3 cells; **(F,G)** glucose uptake **(F)** and lactate production **(G)** in H1299 and Calu-3 cells; **(H)** colony formation ability of H1299 and Calu-3 cells examined by the colony formation assay; **(I)** apoptotic bodies in H1299 and Calu-3 cells examined by the TUNEL assay. Differences were compared by two-way ANOVA **(A–D,F–I)**. All data were presented as the mean ± SD from three independent experiments. **p < 0.01.

To validate the function of MKP1 in NSCLC, oe-MKP1 was further introduced into H1299 and Calu-3 cells by circ_0006349 silencing, and the transfection efficacy was confirmed by RT-qPCR and western blot analysis (Figures 7A,B). After that, it was found that the initially reduced proliferation ability of cells was restored following MKP1 upregulation (Figure 7C), while the apoptosis rate of cells declined (Figure 7D). Nevertheless, the reduced ECAR, glycolysis, glucose uptake, and lactate production in cells suppressed by si-circ_0006349 silencing were recovered following MKP1 upregulation (Figures 7E–G). Likewise, further overexpression of MKP1 increased the number of cell colonies and reduced the TUNEL-positive rate in H1299 and Calu-3 cells (Figures 7H,I). These results indicated that MKP1 rescued the malignant behaviors of NSCLC cells inhibited by si-circ_0006349. In addition, the sole role of MKP1 in cells was examined by transfecting MKP1 siRNA into H1299 and Calu-3 cells. The transfection efficacy was validated by RT-qPCR and western blot assays again (Supplementary Figures 1A,B). Silencing MKP1 significantly reduced the proliferation (Supplementary Figures 1C,D) but increased the apoptosis of cancer cells (Supplementary Figures 1E,F).

## Discussion

Non-small-cell lung cancer (NSCLC) remains a huge health concern worldwide. Most patients with NSCLC are diagnosed at advanced stages due to limited screening programs and late clinical presentations, leading to an unfavorable prognosis (Gridelli et al., 2015). Cancer cells produce energy from lactate fermentation after glycolysis, therefore obtaining more ATP for metabolic activities and proliferation (Abbaszadeh et al., 2020). In the present study, we revealed a novel ceRNA network involving circ_0006349, miR-98, and MKP1 that was possibly implicated in glycolysis, proliferation, and apoptosis in NSCLC.

Comprehensive bioinformatic analyses are very helpful in monitoring gene expression or protein functions in different physiological and pathological situations (Zhou et al., 2020). In this study, using the GEO GSE datasets, circ_0006349 was indicated as the most significantly upregulated circRNA in NSCLC samples. Increasing evidence suggests that circRNAs are not a simple side product of splicing but a new subtype of ncRNAs in higher eukaryotes that are emerging as regulators or biomarkers for human cancers (Ou et al., 2017; Zhao and Shen, 2017; Chen and Huang, 2018). This is also true for NSCLC (Chen et al., 2018; Li et al., 2019). In most cases, circRNAs exert their functions by interacting with miRNAs. For example, circ_HIPK3 plays an oncogenic role in LC by suppressing miR-124 (Yu et al., 2018). Likewise, circ_103809 was demonstrated to promote LC progression by sequestering miR-4302 and encouraging ZNF121-dependent MYC expression (Liu W. et al., 2018). In some cases, circRNAs may also work as tumor suppressors. For instance, circ_0007059 was found to block epithelial-mesenchymal transition and proliferation of LC cells by suppressing miR-378 (Gao et al., 2019). Circ_0001649 inhibited NSCLC progression by sponging miR-331-3p and miR-338-5p, which served as a candidate prognostic biomarker indicating better prognosis and reduced tumor stage and lymph node metastasis in patients (Liu T. et al., 2018). Here, this study identified that high expression of circ_0006349 was correlated with positive lymph node metastasis, increased tumor grades, and poor tumor differentiation. Furthermore, downregulation of circ_0006349 reduced proliferation, glycolysis, glucose uptake, and lactate production in H1299 and Calu-3 cells. These results preliminarily revealed an oncogenic function of circ_0006349 in NSCLC.

CircRNAs frequently exert their functions by sponging miRNAs. After the identification of the cytoplasmic localization of circ_0006349 in cells, we predicted the possible target miRNAs of circ_000634, among which miR-98 was predicted to be downregulated in NSCLC samples according to the data in the GSE29250 dataset. miR-98 has been well documented as a tumor suppressor. For instance, it targeted IGF1R to suppress the proliferation and invasion of colon cancer cells (Liu et al., 2020). Additionally, miR-98 was found to suppress the “Warburg effect,” namely, glycolysis, glucose uptake, and lactate production in colon cancer cells (Zhu et al., 2017). In LC, downregulation of miR-98-5p by lncRNA SNHG4 triggered the proliferation and aggressiveness of LC cells (Tang et al., 2019). This miRNA was also shown to suppress the malignancy of NSCLC by targeting AGL3 (Ke et al., 2020). In the present study, we found that downregulation of miR-98 led to an increase in glycolysis and proliferation of H1299 and Calu-3 cells that were initially reduced upon circ_0006349 silencing. In addition, miR-98 was poorly expressed in the collected tumor tissues from patients, while higher expression of miR-98 was correlated with better clinical presentations of these patients. This was quite in line with a previous study by Wang et al. (2017), which suggested that poor serum miR-98 indicates unfavorable prognosis in patients with NSCLC.

Following the findings above, we predicted the target mRNAs of miR-98 on the bioinformatics systems StarBase and TargetScan, while the differentially expressed mRNAs between NSCLC tissue and normal samples were screened using a GSE51852 dataset. Among the intersecting mRNAs, MKP1 attracted our attention since it has been reported to improve cisplatin resistance and reduce apoptosis in NSCLC cells (Chattopadhyay et al., 2006). MKP1 mediates dephosphorylation and inactivation of N-terminal c-Jun kinase (JNK) and other apoptosis-related kinases, therefore leading to a reduction in apoptosis (Roos and Kaina, 2006; Cortes-Sempere et al., 2009). In addition, MKP1 was found to promote angiogenesis, invasion, and metastasis in NSCLC (Moncho-Amor et al., 2011). In the present study, high expression of MKP1 was confirmed in tumor tissues collected from NSCLC patients, and overexpression of MKP1 in H1299 and Calu-3 cells increased glycolysis and proliferation of H1299 and Calu-3 cells that were initially suppressed by si-circ_0006349.

In summary, this study demonstrated an oncogenic role of circ_0006349 in NSCLC cells through a ceRNA network involving the miR-98/MKP1 axis. However, the potential involvement of the downstream pathways was not considered in this study. All functional experiments were performed *in vivo*. We would like to validate the functions of these molecules in animal models in the near future. We hope that these findings may offer novel insights into NSCLC management.

## Data Availability Statement

The datasets presented in this study can be found in online repositories. The names of the repository/repositories and accession number(s) can be found in the article/Supplementary Material.

## Ethics Statement

The studies involving human participants were reviewed and approved by the Ethics Committee of Wuxi People’s Hospital. The patients/participants provided their written informed consent to participate in this study.

## Author Contributions

HY and TB designed the study. XF and BY collected and cleaned the data. HZ and RZ reviewed the medical records. CQ, RL, and MY helped in data collection and wrote the manuscript. All authors contributed to the article and approved the submitted version.

## Conflict of Interest

The authors declare that the research was conducted in the absence of any commercial or financial relationships that could be construed as a potential conflict of interest.

## Publisher’s Note

All claims expressed in this article are solely those of the authors and do not necessarily represent those of their affiliated organizations, or those of the publisher, the editors and the reviewers. Any product that may be evaluated in this article, or claim that may be made by its manufacturer, is not guaranteed or endorsed by the publisher.

## Footnotes

1
http://www.bioconductor.org/packages/release/bioc/html/limma.html

2
https://cran.r-project.org/web/packages/pheatmap/

3
http://www.circbank.cn/

4
http://starbase.sysu.edu.cn/

5
http://www.targetscan.org/vert_72/

6
http://bioinformatics.psb.ugent.be/webtools/Venn/
