# Supplementary figures and images for: Circular RNA 0006349 Augments Glycolysis and Malignance of Non-small Cell Lung Cancer Cells Through the microRNA-98/MKP1 Axis
Source: Front Cell Dev Biol. 2021 Sep 17;9:690307. doi: 10.3389/fcell.2021.690307 (PMC8484757; doi:10.3389/fcell.2021.690307)

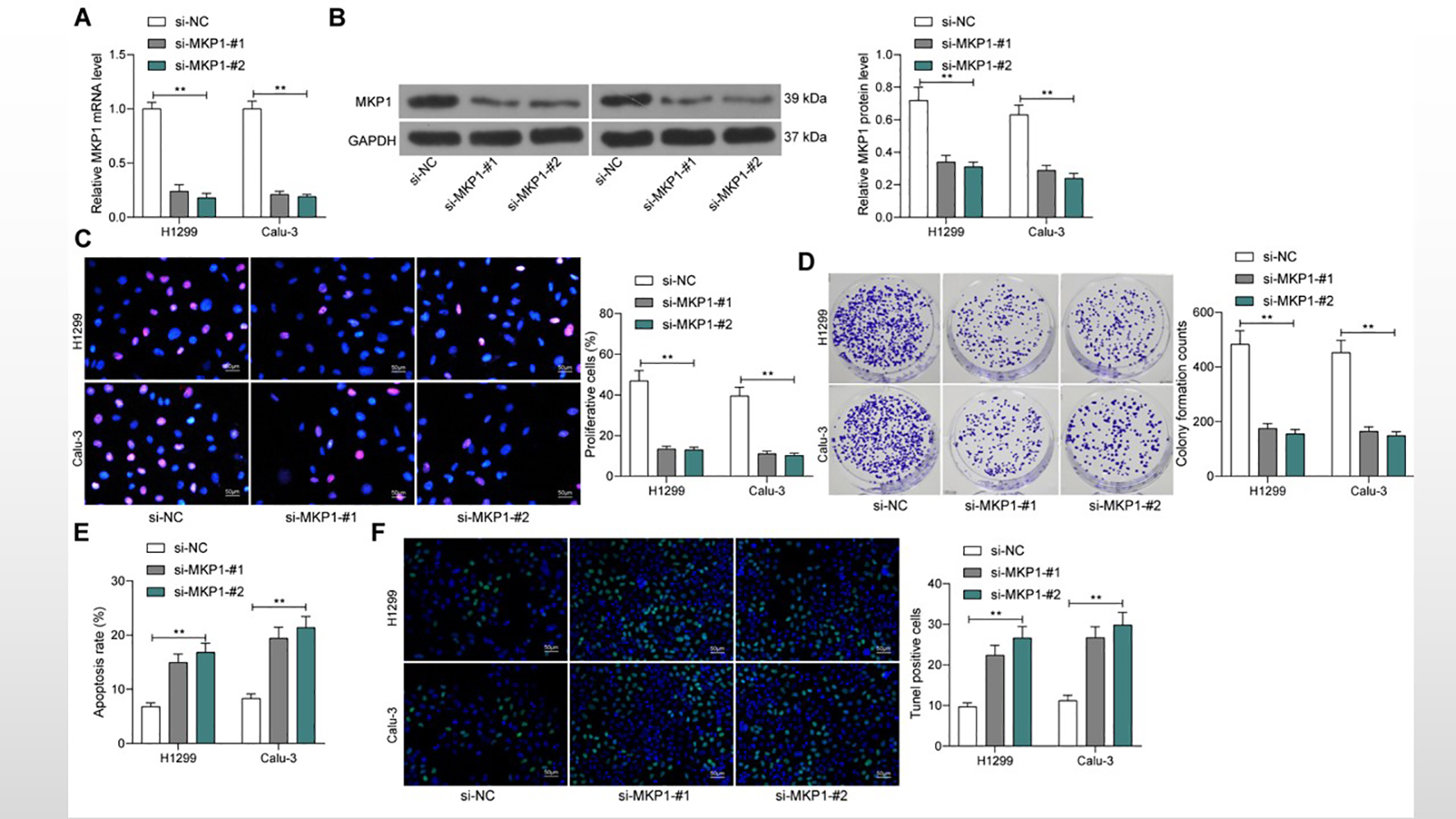

Supplement: Supplementary file 1 [file Image_1.jpg]
